# Parental alienation – a valid experience?

**DOI:** 10.1177/14034948231168978

**Published:** 2023-04-23

**Authors:** Eivind Meland, Dag Furuholmen, David Jahanlu

**Affiliations:** 1 Department of Global Public Health and Primary Care and Bergen Municipality, University of Bergen, Norway; 2 Retired, Nesoddtangen, Norway; 3 Faculty of Health Sciences, Oslo Metropolitan University (OsloMet), Akershus, Norway

**Keywords:** Parental alienation, domestic violence, depression, impaired well-being

## Abstract

**Background:**

The phenomenon of ‘parental alienation’ is controversial and little explored in the Nordic countries. We wanted to investigate whether parental alienation is a valid concept and how it is perceived in a Nordic context.

**Material and method:**

The study was based on an online survey where the participants were self-recruited. We received responses from 1212 participants. Bivariate and multivariate models were used to test the associations between parental alienation and gender, other intimate partner violence, depressive health problems and reduced well-being.

**Results:**

Visitation sabotage and parental alienation are realities for both fathers and mothers. It was most frequently directed at fathers, but such behaviour is not gender specific. Eight different alienation strategies have high internal reliability, and all items contribute to high Cronbach’s alpha. Construct validity was confirmed by the fact that parental alienation is strongly associated with visitation sabotage and with other forms of destructive relational behaviour. Visitation sabotage and false accusations increase gradually with the degree of parental alienation. The construct validity of parental alienation was also confirmed by dose–response associations with both mental ill-health and impaired well-being in adjusted analyses.

**Conclusions:**

**The phenomenon of parental alienation is recognized among parents as a form of harmful behaviour where both mothers and fathers suffer. The construct validity was supported in this study. Such behaviour should be recognized as a form of domestic violence by professional communities in health and social services and be subject to legal action.**

## Introduction

Children who are influenced by parents, therapists or employees in welfare services so that they create an enemy image of the parent(s) with whom they do not have permanent residence may be exposed to a destructive dynamic that, internationally, has the term ‘parental alienation’ [1–3]. A recent literature review describes parental alienation as a complex form of family violence directed at a parent in order to damage their relationship with their own children [3].

The prevalence of parental alienation in the Nordic countries has been only indirectly explored with lost contact between children and biological parents. By the age of 17 years, 12% of the adolescents reported loss of parental contact, mostly with fathers [4]. Another Norwegian study from cross-sectional data over 13 years found that an increasing number of adolescents lost contact, almost exclusively with their fathers [5]. Statistics Norway has estimated that between 26,000 and 46,000 children do not see their fathers during an average month [6].

The public health relevance is firmly documented by a representative study from the USA showing that the phenomenon is widespread, as nearly 10% of the adult population had been exposed to significant parental alienation by their partners [7]. The consequences for children of being exposed to such behaviour by a parent, like other forms of family violence, are large and lifelong, with increased crime, school dropout, substance abuse problems, depression and anxiety as a result. In addition, such experiences in childhood will often lead to the repetition of similar behaviour in adulthood: the alienation ‘infects’ new generations [8].

Whereas previously it was common to talk about a ‘syndrome’ that manifested itself in the child [9], the focus is now on a relationship problem that meets five main criteria [10]:

1. The child avoids, opposes or refuses to have a relationship with a parent.
2. The child has previously had a positive relationship with the parent who is now alienated.
3. The alienated parent has not subjected the child to abuse or neglect.
4. The favoured parent employs multiple alienating strategies and methods.
5. The child shows signs of behavioural disturbance that indicate alienation.

The alienating parent uses manipulative parenting behaviour to change the child’s thoughts and feelings about the other parent. For example, convincing the child that the other is a bad parent and a bad person who does not deserve their love and respect, and promotes negative themes that highlight the other parent’s real, exaggerated or imagined shortcomings and flaws. Unsubstantiated and false allegations of forms of abuse can be used by one parent to gain advantage over the other parent – allegations of domestic violence, physical and sexual abuse of children, or neglect [11]. Parental alienation is manifested in the child with hostile feelings and cognitions against a parent, often with lack of ambivalence and appropriate justifications [3].

In Nordic countries, parts of the academic community have been reluctant to recognize the phenomenon as harmful to children’s health and functioning. Some researchers still claim that parental alienation lacks construct validity [12]. The Ministry of Children and Equality in Norway has warned against having confidence in a party to the court who refers to parental alienation. The Ministry has taken the view that parental alienation is not scientifically documented, and that it may lead to victims of violence and abuse being distrusted [13].

Research on health consequences is most closely related to harmful effects on children, and on adults who have been exposed to such impacts as children. Consistent findings with impaired mental and social health confirm that this phenomenon has construct validity [3]. An increasing body of research is now also linked to parents who are victims of alienation by the other parent or by public officials [14,15]. Alienated parents often have significant anxiety, depression, stress and physical symptoms. They may feel powerless, hopeless and socially isolated. Several studies found that they felt they had lost part of their identity due to losing their role as a parent. Four studies reported high levels of suicidality among alienated parents [11]. This, too, confirms that this is a phenomenon that has construct validity and substantially impacts public health.

Over the past couple of years, some key professional bodies in Norway have also recognized that this phenomenon entails a major health risk that must be taken seriously by health care institutions as well as judicial bodies [16,17]. Historically, parental alienation has been viewed with suspicion by advocates fighting domestic violence against women. Some spokespersons for this important campaign still hold the opinion that parental alienation is a fake excuse from male perpetrators of domestic violence, whereas some now realize that parental alienation is not a gender-specific phenomenon as it is also directed against mothers.

However, we have not been able to identify any studies from Nordic countries investigating the prevalence or health consequences of parental alienation. With data from an online survey, we therefore set ourselves the goal of investigating:

- Whether parental alienation is a phenomenon that is recognizable by parents;
- Whether parental alienation can be conceptualized as a valid construct;
- The extent to which parental alienation is a gender-specific phenomenon.

## Material and method

Two of the authors (DF and EM) were responsible for the preparation of questions in this online survey on family and divorce experiences. The study was based on material where the participants were self-recruited and presented with a questionnaire on a professional survey platform hosted by an online management system, SurveyXact™ (Ramböll Management Consulting, Oslo, Norway). The survey was widely shared, mainly by word of mouth, Twitter, Facebook sites and a wide selection of Norwegian Facebook groups that were expected to be relevant for this topic. The invitation stated that the survey intended to examine quality of life, psychological health, and child-care cooperation among parents after divorce and cohabitation break-up. Prevalence data cannot be transferred to the general population. On the other hand, it is important to say that a significant number of participants reported currently stable and unproblematic cohabitations without custody conflicts. The questionnaire is presented as supplemental material on the net.

### Participants

Twelve hundred and twelve people responded (68% male and 32% female). Of those, 820 answered the survey completely. All answers were used in the analyses. Table I shows the participants, with separate columns for men and women with *p*-values from cross tabulation Chi-squared tests.

**Table I.** Participants who responded to an online survey on cohabitation, marital conflicts, parental alienation, depressive health problems, and impaired well-being according to gender, with *p*-values from Chi-squared tests based on cross-tabulation.

Variable	Gender		*p*-value
	Men	Women	
**Highest attained education**			
Primary and secondary school	180 (22.8%)	49 (13.0%)	<0.001
Vocational education	172 (21.8%)	40 (10.6%)	
University college and university	437 (54.4%)	289 (76.4%)	
Total	789 (100%)	378 (100%)	
**Income level**			
Up to NOK400,000	117 (14.7%)	60 (15.9%)	0.10
NOK401,000 to 600,000	259 (32.6%)	144 (38.1%)	
NOK601,000 or more	418 (52.6%)	174 (46.0%)	
Total	794 (100%)	378 (100%)	
**Age, grouped**			
30 and lower	40 (5.0%)	13 (3.4%)	0.515
31–40	229 (28.7%)	113 (29.7%)	
41–50	318 (39.8%)	153 (40.3%)	
51–60	165 (20.6%)	85 (22.4%)	
Over 61	47 (5.9%)	16 (4.2%)	
Total	799 (100%)	380 (100%)	
**Marital status**			
Married or cohabiting	247 (32.3%)	100 (27.2%)	<0.001
Divorced or separated	247 (32.3%)	168 (45.7%)	
Single or widowed	270 (35.3%)	100 (27.2%)	
Total	464 (100%)	368 (100%)	
**Depressive symptoms**			
Without depressive symptoms	363 (45.2%)	276 (72.1%)	<0.001
Mild depressive symptoms	235 (29.3%)	50 (13.1%)	
Moderate and pronounced depressive symptoms	205 (25.5%)	57 (14.8%)	
Total	803 (100%)	383 (100%)	
**Well-being**			
Satisfactory	151 (22.0%)	95 (35.4%)	<0.001
Not good	260 (37.9%)	96 (35.8%)	
Bad	275 (40.1%)	77 (28.8%)	
Total	803 (100%)	383 (100%)	

χ^2^(11) = 192.85 *p* < 0.001; McFadden *R*^2^ = 0.12.

### Dependent and independent variables

Parental alienation was constructed on the basis of eight dichotomous individual variables with known alienation strategies from the literature [8]. We constructed a composite variable as an average of these eight individual variables (range 0–1). A composite variable for depressive health problems was constructed on the basis of eight individual questions taken from the Montgomery Aasberg depression rating scale [18]. The responses were ranked on a Likert scale from 1 (not experienced) to 5 (experienced to a very large extent). A composite variable was constructed as a mean value for those who had four or more valid answers (range 1–5).

Similarly, we constructed a composite variable for reduced well-being (affective quality of life) based on individual questions recommended in the literature [19]. The responses were also ranked on a Likert scale from 1 (good well-being) to 4 (very reduced well-being). The mean of the aggregate variable was calculated if two or more responses were valid. Cronbach’s alpha was satisfactory for both aggregate variables (0.83 and 0.89).

The questionnaire included questions about gender, age in 10-year categories, education and income level. We had separate questions related to other forms of destructive relational behaviour: visitation sabotage, unfounded accusations, threats, and psychological and physical violence. These questions were answered both with yes/no and with frequencies. Table II shows how the responses were grouped according to frequency and severity.

**Table II.** Parental alienation and other manifestations of relational conflicts by gender, analysed with independent *t*-tests (supplemented by non-parametric test) and Chi-squared test from cross-tabulated analyses.

Variables	Gender		*p*-value
	Women	Men	
Parental alienation, mean (SD)	0.11 (0.21)	0.25 (0.29)	<0.001
Parental alienation, average rank^a^	476.9	649.1	<0.001
**Visitation sabotage**			
None	223 (85.1%)	295 (53.6%)	<0.001
1–5 times per year	9 (3.4%)	97 (15.3%)	
1–5 times per month	7 (2.7%)	65 (10.2%)	
Continuous	23 (8.8%)	179 (28.1%)	
Total	262 (100%)	636 (100%)	
**Physical violence**			
None	255 (93.0%)	631 (91.6%)	0.19
Few cases	13 (4.7%)	25 (3.6%)	
More times and rougher	6 (2.2%)	33 (4.8%)	
Total	274 (100%)	689 (100%)	
**Unsubstantiated accusations**			
None	346 (90.3%)	477 (59.4%)	<0.001
Few cases	28 (7.3%)	163 (20.3%)	
More and more serious accusations	9 (2.4%)	163 (20.3%)	
Total	383 (100%)	803 (100%)	
**Psychological violence**			
None	288 (75.3%)	518 (64.5%)	0.002
Few cases	45 (11.7%)	143 (17.8%)	
Multiple cases	35 (9.1%)	88 (11.0%)	
More and more serious cases	15 (3.9%)	54 (6.7%)	
Total	383 (100%)	803 (100%)	

a Mann–Whitney *U*-test.

### Statistics

Simple descriptive statistics with frequencies and averages with standard deviation (SD) for continuous variables according to gender with associated *p*-values either from independent *t*-tests or Chi-squared tests from cross-tabulation are presented. We used one-way analysis of variance (ANOVA) when examining the extent to which there was an association between other forms of relational abuse and parental alienation.

The association between depressive health problems and parental alienation had an unsatisfactory ‘fit’ regarding ordinal regression. We therefore analysed this association first with logistic regression (none versus one or more depressive symptoms), and then with linear regression in those who reported depressive health complaints.

We conducted stratified analyses based on gender to investigate whether men and women had a different association between parental alienation on the one hand and depression and reduced well-being on the other. We found overlapping confidence intervals (CIs). We therefore included gender only as an adjusting variable in the multivariate analyses.

Levene’s test was used to investigate variance homogeneity when *t*-test and one-way ANOVA were used. When necessary, for parametric tests we checked the assumption of normal distribution by either Shapiro–Wilk or Q–Q scatterplot, depending on the size of subgroups. Non-parametric tests were applied when we could not reach the necessary assumptions for parametric tests. When comparing the means of more than two subgroups with one-way ANOVA, the assumption of homogeneity of variances were assessed by Levene’s test, and in case the homogeneity was violated, instead of Bonferroni’s post-hoc table, the results were reported from Games–Howell’s analysis.

In the multivariate analyses (Tables IV and V), we investigated whether multi-collinearity could make the models non-valid by calculating the variance-inflation factor. The Box–Tidwell approach was used to judge linear correlation. We judged whether the multivariate models were valid using the Hosmer and Lemeshow goodness-of-fit test (logistic regression) or the Pearson goodness-of-fit test (ordinal regression). In addition, we investigated whether the levels in the ordinal regression analysis were proportional using the likelihood ratio test.

### Ethics

The study was submitted to the Regional Committee for Medical and Health Research Ethics West, and it was exempted from ethical clearance as only anonymous data were collected. The data collection system did not collect IP-addresses, making the survey anonymous.

## Results

Marital status was reported as 30% married or cohabiting, 36% divorced and 31% single. As many as 44% of the participants experienced visitation sabotage. Half of the targeted group (22%) experienced continuous sabotage. Most men had such experiences, but a not insignificant number of women (46 of 269) also reported sabotage. Tables I and II show a descriptive overview of the participants by gender with Chi-squared *p*-values from cross-tabulation or independent *t*-tests (and a Mann–Whitney *U*-test) for comparison for continuous variables.

Table I shows that the participants had a relatively high educational attainment, and that women had significantly better education than men and earn equally well. Men are more likely to live alone and have more depressive symptoms and a lower degree of well-being than women. Table II shows that men and women reported physical violence rarely, but equally often. Men experienced parental alienation, psychological violence, unfounded accusations, and sabotage significantly more often than women.

Marital status (married and cohabiting compared with divorced or separated and single or widowed) had only a borderline significant association (Pearson Chi-squared test, *p* = 0.06) with whether one parent had used alienating behaviour toward the other. Average alienation scores were similar across all marital status groups (not shown in Table II).

In Table III, we have grouped the relational conflicts into three levels and performed one-way ANOVA analyses to explore the associations between these and parental alienation. Table III shows that there is a strong association between the degree of visitation sabotage and unfounded accusations on the one hand and parental alienation on the other. The presence of any form of psychological violence is associated with increased parental alienation. Table III also reveals that physical violence with greater frequency and severity is also statistically significantly associated with parental alienation.

**Table III.** Associations between parental alienation (mean) and other relational abuse (categorized into three levels) analysed using one-way analysis of variance (ANOVA) with Bonferroni or Games–Howell post-hoc correction.

Variables	Comparison of categories Level 0–2 (*n*)		Mean difference (*p*-value)
**Psychological violence**	0 (*n*= 831)	1 (*n*=189)	−0.25 (<0.001^a^)
	0 (*n*=831)	2 (*n*=192)	−0.25 (<0.001^a^)
	1 (*n*=189)	2 (*n*=192)	−0.00 (0.996)
**False accusations**	0 (*n*=849)	1 (*n*=191)	−0.26 (<0.001^a^)
	0 (*n*=849)	2 (*n*=172)	−0.41 (<0.001^a^)
	1 (*n*=191)	2 (*n*=172)	−0.15 (<0.001^a^)
**Physical violence**	0 (*n*=886)	1 (*n*=38)	−0.03 (1.0^a^)
	0 (*n*=886)	2 (*n*=39)	−0.22 (<0.001^a^)
	1 (*n*=38)	2 (*n*=39)	−0.19 (0.014^a^)
**Visitation sabotage**	0 (*n*=524)	1 (*n*=179)	−0.29 (<0.001^a^)
	0 (*n*=524)	2 (*n*=203)	−0.42 (<0.001^a^)
	1 (*n*=179)	2 (*n*=203)	−0.14 (<0.001^a^)

a *p*-value from ANOVA test with Bonferroni or Games–Howell post-hoc correction.

Reliability testing of the eight alienation strategies showed high Cronbach’s alpha (0.85). All eight items contributed to increased reliability.

Table IV shows the association between parental alienation and depressive health complaints (no symptoms in relation to all degrees of depressive symptoms). We have adjusted for gender, age, education level and income. The odds ratio (OR) tells us that in relation to no alienation, odds increase by more than seven times when the average value increases to 1 (presence of all characteristics of alienation). We have supplemented this analysis with multivariate linear regression in those who reported the presence of one or more depressive symptoms (not shown in Table IV). The standardized regression coefficient was 0.29 (*p*<0.001) when controlling for the same adjustment variables as in Table IV. This indicates that there is a linear relationship between parental alienation, so that when alienation increases with one SD, depressive symptoms increase by 0.29 SD.

**Table IV.** Binary logistic regression, adjusted for gender, age, educational attainment and income, showing the associations between parental alienation and depressive health problems (no symptoms in relation to all degrees of depressive symptoms). The model had a significant association with the presence of depressive symptoms (*p*<0.001).

Variables	B	*p*	OR	95.0% CI
**Parental alienation**	2.01	<0.001	7.47	(4.51, 12.37)
**Gender, men**	0.90	<0.001	2.47	(1.84, 3.31)
**Age group 31–40**	0.33	0.32	1.39	(0.72, 2.68)
**Age group 41–50**	0.33	0.32	1.39	(0.73, 2.67)
**Age group 51–60**	−0.042	0.91	0.96	(0.48, 1.90)
**Age group over 61**	−0.94	0.04	0.39	(0.16, 0.96)
**Level of education, vocational education**	0.13	0.53	1.14	(0.76, 1.73)
**Education level, university/university college**	−0.36	0.05	0.70	(0.50, 0.99)
**Income NOK401,000–600,000**	0.02	0.91	1.02	(0.69, 1.53)
**Income NOK601,000–800,000**	−0.22	0.30	0.80	(0.52, 1.22)
**Income over NOK801,000**	−0.09	0.71	0.92	(0.58, 1.45)

OR: odds ratio; CI: confidence interval.

Table V shows the results of ordinal logistic regression for the association between parental alienation and reduced emotional well-being. We divided the participants into three levels of well-being. Those who reported no reduction in well-being (*n* = 246) were compared with two equally sized groups with declining well-being. Here, too, we adjusted for the same factors as in Table IV. The results show that odds increased by close to three times to experience reduced well-being when parental alienation increases from 0 to 1. The increase is gradual in that the OR is equal from ‘satisfactory’ to ‘not good’ and from ‘not good’ to ‘bad’.

**Table V.** Ordinal logistic regression, adjusted for gender, age, educational attainment and income, showing the associations between parental alienation and impaired well-being (impaired well-being is categorized into three levels). The model was significantly associated with the presence of reduced well-being (*p*<0.001).^a^

Variables	B	*p*	OR	95.0% CI
**Parental alienation**	1.05	<0.001	2.86	(1.82, 4.49)
**Gender, men**	0.52	<0.001	1.68	(1.26, 2.24)
**Age group 31–40**	−0.28	0.38	0.76	(0.41, 1.41)
**Age group 41–50**	−0.19	0.55	0.83	(0.45, 1.53)
**Age group 51–60**	−0.39	0.24	0.68	(0.35, 1.29)
**Age group over 61**	−1.51	0.002	0.22	(0.09, 0.58)
**Level of education, vocational education**	0.31	0.11	1.40	(0.93, 2.01)
**Education level, university/university college**	0.10	0.54	1.11	(0.80, 1.55)
**Income NOK401,000–600,000**	−0.36	0.07	0.70	(0.47, 1.02)
**Income NOK601,000–800,000**	−0.47	0.02	0.62	(0.42, 0.94)
**Income over NOK801,000**	−0.49	0.03	0.61	(0.39, 0.95)

a Proportional odds assumption was met (non-significant likelihood ratio; *p*=0.450).

OR: odds ratio; CI: confidence interval.

## Discussion

The construct validity of parental alienation was confirmed by the existence of dose–response associations with other forms of relational violence, mental ill-health and impaired well-being. The associations were also clear when we adjusted for relevant confounding variables. Construct validity was also confirmed by the associations between parental alienation and other destructive relational behaviour. Visitation sabotage and false accusations increase gradually with the degree of parental alienation. Furthermore, the survey shows that visitation sabotage and parental alienation are realities for both fathers and mothers. Parental alienation and visitation sabotage were most frequently directed at fathers, but such behaviour was not gender specific.

We chose depression and reduced emotional well-being as our main outcomes for checking construct validity, as these are core elements in the reactions experienced by targeted parents [14]. The present study supports our investigation, that parental alienation demonstrates construct validity in a Nordic setting, opposing what some researchers maintain [12]. A growing body of research literature confirms that the phenomenon is well founded and validated [8,11]. However, recurrent misinformation in the literature is still promoted by strong advocacy groups. Their claims have been subjected to conscientious evaluation in a recent paper [20].

In the research literature, we find similar gender inequalities as in this study [21]. The reason why more men are victims of such behaviour is explained by more mothers having main or sole care of children after divorce and break-ups. The differences are almost erased when controlling for children’s custody arrangements. There are some gender differences about the methods used in the alienation processes in that women use indirect methods and emotional control to a greater extent than men [21]. A few studies find gender differences in the use of false claims, where fathers are the targets of false allegations of sexual abuse, and mothers are more often victims of false claims of neglect [18,19,22,23].

We found no association between parental alienation and marital status. This finding contradicts findings in the literature, which almost without exception reveal that it is a phenomenon that develops in the wake of divorce and break-up [3]. We asked about current status and not about experience of divorce/break-up. It may therefore be somewhat random if those who have experienced a break-up reported a re-established relationship, single living status or status as divorced.

We found no statistically significant gender differences when it came to being exposed to physical violence. However, few women were exposed to physical violence, and the statistical power was low concerning this finding. Therefore, we cannot rule out gender differences based on this study alone. In many contexts, we have long had a strong gender-specific focus on domestic violence. The reason may be that there is a big difference in the extent to which male and female victims report this [24]. A review article in which several representative population studies were included showed that there were few or no gender differences [25].

Women more often expose their partners to verbal aggression and harassment in the form of criticism and disparagement [24]. In the present study, this was also evident in the fact that men more frequently than women experienced visitation sabotage, unfounded accusations, psychological violence and parental alienation.

There is a consistent finding in the literature that parents, regardless of gender, use unsubstantiated claims of abuse to gain advantage over the other parent in connection with break-ups. There may be allegations of domestic violence, physical and sexual abuse of children, neglect or parental alienation. Common themes are that the other parent is to blame for the break-up in the family or that the other parent is dangerous, does not really love their children or prioritizes other things – such as work, money and a new partner – over the children [11]. The strong association between parental alienation and false accusations in our study confirmed that this is a characteristic feature. That there is also a linear relationship with visitation sabotage is no surprise. Preventing and denying access is one of the main criteria for parental alienation [3,11].

The effect of parental alienation on children’s mental and social health has been thoroughly documented [2,3]. Harmful effects on targeted parents have been increasingly investigated. The injuries were consistent with victims of other forms of coercive control in one study [26]. Alienated parents have significantly more anxiety, depression, stress and physical symptoms, and they report powerlessness, hopelessness and social isolation. Several studies found that victims of such alienation felt that they had lost part of their identity owing to losing their role as a parent, and they felt intense negative emotions related to the loss of their children. Several studies have found a strong association with suicidality [27]. This is consistent with our findings, with a strong association with depressive health problems and reduced quality of life and well-being.

We would like to emphasize that it is important that therapists in the health service and in child and family welfare services and professional groups responsible for legal protection familiarize themselves with and assess the possibility that parental alienation may exist. A previous article in the *Journal of the Norwegian Medical Association* demonstrated how a lack of professional competence can reinforce alienation and further harm children [28]. In recent years, the Human Rights Court verdicts against Norway have almost without exception been based on excessively strict restrictions on visitation between children and biological parents [29].

This research field is largely neglected in the Nordic countries. It is difficult to understand why. With large, high quality epidemiologic data we should be able to penetrate these phenomena in greater detail and also check how parental alienation may ‘infect’ new generations. This would add importance to the public health relevance of the phenomenon.

### Weaknesses and strengths

The participants in the study were self-recruited with invitations via the internet. We cannot claim representativeness. The age distribution corresponds with the average Norwegian population, although the oldest age group is under-represented as compared with the general population [30]. More men than women participated. The respondents’ educational attainment is well above the Norwegian average [31].

The reason why more men than women responded is probably an expression of the fact that more men experience these forms of violence in close relationships. The prevalence of parental alienation and visitation sabotage is higher in our study than in the general population but does not differ radically from other studies in the general population [4,7]. The study was a cross-sectional study using a common questionnaire. Dose–response associations may indicate causal relationships, but we acknowledge that such associations may be influenced by ‘common method bias’.

The strength of the study is that we report findings from a field that has been inadequately researched in the Nordic countries. We have mapped several destructive relational behaviours and documented that parental alienation is recognizable by parents, and that it is a phenomenon that has a high degree of construct validity.

### Interpretation

The phenomenon of parental alienation is recognized among parents as a form of harmful behaviour where both mothers and fathers suffer. The construct validity was thus supported. Such behaviour should be recognized as a form of domestic violence by professional communities in health and social services and be subject to legal action.

## Supplemental Material

sj-docx-1-sjp-10.1177_14034948231168978 – Supplemental material for Parental alienation – a valid experience? Supplemental material, sj-docx-1-sjp-10.1177_14034948231168978 for Parental alienation – a valid experience? by Eivind Meland, Dag Furuholmen and David Jahanlu in Scandinavian Journal of Public Health
