# Supplementary material for: Parental alienation – a valid experience?
Source: Scand J Public Health. 2023 Apr 23;52(5):598–606. doi: 10.1177/14034948231168978 (PMC11292963; doi:10.1177/14034948231168978)
Supplement: sj-docx-1-sjp-10.1177_14034948231168978 – Supplemental material for Parental alienation – a valid experience? [file sj-docx-1-sjp-10.1177_14034948231168978.docx]

**Appendix to the internet (supplementary)**

SURVEY 2021 ON QUALITY OF LIFE AFTER BREAKUP.

We hope you can help us answer this survey so that we learn more about how people feel when they go through a breakup. We also want to map the relationships between parents' quality of life and mental health, based on the collaborative situation regarding joint children in the breakup. We believe that there is a particular lack of knowledge about men's life situation after a break-up, but we also want answers from women about their life situation.

The survey is anonymous, which ensures that the answers cannot be linked to the individual respondent or IP addresses. This applies to all *SurveyXact* users regardless of their access/user role, as well as *SurveyXact's* employees and support service.

The survey is conducted under the auspices of *MannsForum* and penned by Prof. Emeritus Eivind Meland and psychiatrist Dag Furuholmen.
The survey takes about 8 minutes. Thank you for taking your time!

I hereby give permission for my answers to be registered anonymously in a database and used for research purposes.

(1) Yes

(2) No

Gender

(1) Male

(2) Female

(3) Other/Do not wish to state

Age

(1) Under 21

(2) 21-30

(3) 31-40

(4) 41-50

(5) 51-60

(6) 61-70

(7) Over 70

(8) Do not wish to disclose

Education level

(1) Primary school

(2) High School

(3) Professional education

(4) College/Bachelor

(5) University/Master and equivalent

(6) Other/Do not wish to state

Job

(1) Full-time

(2) Part-time

(3) Student

(4) Unemployed

(5) On sick leave

(6) Disabled

(7) Other/Do not wish to state

Gross income (income before tax/contributions)

(1) Up to 200,000/year

(2) 201-400,000/year

(3) 401-600,000/year

(4) 601-800.000/year

(5) 801-1 million/year

(6) Over 1 million/year

(7) Other/Do not wish to state

Dwelling

(1) Own dwelling with its own room for children

(2) Own dwelling without its own room for children

(3) Rented dwelling with its own room for children

(4) Rented housing without its own room for children

(5) Homeless

(6) Borrowing/renting housing for visitation

(7) Other/Do not wish to state

Marital status

(1) Married

(2) Cohabiting

(3) Separated/separated

(4) Single

(5) Widow/widower/surviving partner

(6) Other/do not wish to state

How many children do you have?

|  | 0 | 1 | 2 | 3 | 4 | 5 | More than 5 |
| --- | --- | --- | --- | --- | --- | --- | --- |
| Boy | (1) 🔾 | (7) 🔾 | (2) 🔾 | (3) 🔾 | (4) 🔾 | (5) 🔾 | (6) 🔾 |
| Girl | (1) 🔾 | (7) 🔾 | (2) 🔾 | (3) 🔾 | (4) 🔾 | (5) 🔾 | (6) 🔾 |

**Do you have legal decision-making authority (resident parent)**

(1) Has decision-making authority (the child has permanent residence with me)

(2) Has shared decision-making authority (shared residence)

(3) Does not have decision-making authority (the child has a permanent residence with an ex-partner)

(4)I have a permanent residence for one or more children and the ex-partner has a permanent residence for one or more children.

(5) Other/Do not wish to state

What do you pay per month in contributions + expenses for visitation (travel expenses, etc.)

(1) Up to 1,000

(2) 1.001-2.000 / month

(3) 2.001-4.000 / month

(4) 4.001-6.000 / month

(5) 6.001-8.000 / month

(6) 8.001-10.000 / month

(7) 10.001-12.000 / month

(8) Over 12,000/ month

(9) Other/Do not wish to state

Number of children you pay for

(1) 1

(2) 2

(3) 3

(4) 4

(5) More

(6) Other/Do not wish to state

Spending time with your own children

(1) Living with the children full-time

(2) Togetherness 61% or more

(3) Togetherness 51 - 60%

(4) Togetherness 41-50%

(5) Togetherness 31-40%

(6) Togetherness 21-30%

(7) Togetherness 11-20%

(8) Togetherness 10% or less

(9) Supervised visitation only

(10) I do not have contact with the children

(11) Other/Do not wish to state

Is your contact with your own children sabotaged?

(1) Yes

(2) No

How often are your interactions with your own children sabotaged?

(1) Sabotaged 1-5 times/year

(2) Sabotaged 1-5 times/ month

(3) Sabotaged 1-5 times/week

(4) Sabotaging togetherness continuously

(5) Other/Do not wish to state

Have you had contact with child welfare services?

(1) Yes

(2) No

I have experienced the contact with the child welfare services as

(1) Very difficult

(2) Difficult

(3) Neither nor

(4) Good

(5) Very good

(6) Other/Do not want to state _____

Have you had contact with family counselling?

(1) Yes

(2) No

I have experienced the contact with the family counselling service as

(1) Very difficult

(2) Difficult

(3) Neither nor

(4) Good

(5) Very good

(6) Other/Do not want to state

Have you had contact with the judiciary in connection with divorce?

(1) Yes

(2) No

I have experienced contact with the judiciary as

(1) Very difficult

(2) Difficult

(3) Neither nor

(4) Good

(5) Very good

(6) Other/Do not wish to state

I have experienced the contact with and information from kindergartens such as

(1) Very difficult

(2) Difficult

(3) Neither nor

(4) Good

(5) Very good

(6) Other/Do not wish to state

I have experienced the contact with and information from school as

(1) Very difficult

(2) Difficult

(3) Neither nor

(4) Good

(5) Very good

(6) Other/Do not wish to state

Have you experienced unsubstantiated accusations from your partner?

(1) Yes

(2) No

What unsubstantiated accusations have you experienced?
More choices possible

(1) Allegations of threats of violence or violence against a partner

(2) Allegations of threats or violence against the child(s)

(3) Allegations of sexual abuse against partner

(4) Allegations of sexual abuse of the child(s)

(5) Have unfounded allegations been reported to the CPS

(6) Have unsubstantiated allegations been reported to the police/judiciary

(7) Other/Do not want to state

Have you experienced that your partner has tried to turn your children against you and create hostility?

(1) Yes

(2) No

How have you experienced your partner trying to turn your children against you and create hostility?

More choices possible

(1) Manipulates, boycotts, or muddles the contact between you and your child

(2) You are described as naughty, dangerous, or not fond of the child

(3) Forcing the child to choose one parent

(4) Threatens children with loss of love and/or loyalty

(5) Purges image and common history

(6) Changes children's last name and or refers to me by first name instead of "mother/father"

(7) The child no longer gets to see grandparents or relatives in your family

(8) Spying, controlling, or using children, etc. for this purpose

(9) Other/Do not want to state

Do you find that the child/children are acting hostile or have suddenly become afraid of you?

(1) Yes

(2) No

If you experience skepticism or hostility from your child/children after a breakup which description is best suited?

(1) Mild: The child shows a certain degree of negative attitudes towards you and a certain resistance to contact

(2) Moderate: The child shows more pronounced negative attitudes and clearly expresses a reluctance to visit, but accepts under protest

(3) Serious: The child expresses very negative beliefs, including outright hatred or fear of me as a parent.

(4) Oppose any form of contact

(5) Other/Do not want to state

Have you had a loving relationship in the past, then suddenly completely transformed with unclear/unlikely justification?

(1) Yes

(2) No

Have you been subjected to threats or psychological violence from your partner in the past year?

(1) Yes

(2) No

How often have you been subjected to critical comments and derogatory designations of you as a partner?

(1) 1-5 times a year

(2) 1-5 times a month

(3) 6 or more times a month

(4) Other/Wishes not stated

How often have you been subjected to abusive, derogatory terms or threats of, for example, revenge?

(1) 1-5 times a year

(2) 1-5 times a month

(3) 6 or more times a month

(4) Other, describe: _____

Have you been subjected to violence from your partner/ex-partner in the past year?

(1) Yes

(2) No

How often have you been exposed to violence from your partner/ex-partner in the past year?

|  | 1-5 times | 6-10 times | More than 10 times | None |
| --- | --- | --- | --- | --- |
| Light violence (pushing, ear ticking, scratching) | (1) 🔾 | (2) 🔾 | (3) 🔾 | (4) 🔾 |
| Threats or psychological violence | (1) 🔾 | (2) 🔾 | (3) 🔾 | (4) 🔾 |
| Aggravated assault | (1) 🔾 | (2) 🔾 | (3) 🔾 | (4) 🔾 |
| Sexual violence | (1) 🔾 | (2) 🔾 | (3) 🔾 | (4) 🔾 |

Other, describe: _____

*Avoid sharing personally identifiable information to ensure anonymity.*

Have you even made threats or psychological violence against your ex-partner in the past year?

(1) Yes

(2) No

How often have you even made threats or psychological violence against your ex-partner in the past year?

|  | 1-5 times | 6-10 times | More than 10 times | None |
| --- | --- | --- | --- | --- |
| Critical comments and derogatory designations of you as a partner | (1) 🔾 | (2) 🔾 | (3) 🔾 | (4) 🔾 |
| Grossly derogatory terms or threats of, for example, revenge | (1) 🔾 | (2) 🔾 | (3) 🔾 | (4) 🔾 |

Other, describe: _____

*Avoid sharing personally identifiable information to ensure your anonymity.*

Have you committed violence against your ex-partner in the past year?

(1) Yes

(2) No

How often have you even exercised violence against your ex-partner in the past year?

|  | 1-5 times | 6-10 times | More than 10 times | None |
| --- | --- | --- | --- | --- |
| Light violence (pushing, ear ticking, scratching) | (1) 🔾 | (2) 🔾 | (3) 🔾 | (4) 🔾 |
| Aggravated assault | (1) 🔾 | (2) 🔾 | (3) 🔾 | (4) 🔾 |
| Sexual violence | (1) 🔾 | (2) 🔾 | (3) 🔾 | (4) 🔾 |

Other, describe: _____

*Avoid sharing personally identifiable information to ensure your anonymity.*

**Part 2. Own experience of quality-of-life health**

1. When you think about how you feel at the moment, you are

(1) Mostly satisfied

(2) Somewhat satisfied

(3) Somewhat dissatisfied

(4) Mostly dissatisfied

(5) Other/Do not want to state

2. How obvious do you feel mostly at the moment?

(1) Obvious

(2) Pretty obvious

(3) Something tired and tired

(4) Mostly tired and tired

(5) Other/Do not want to state

3. What is your overall mood/mood like in everyday life at the moment?

(1) Is in a good mood

(2) Is in a good mood

(3) Often depressed or sad

(4) Is mostly depressed or sad

(5) Other/Do not want to state

4. How would you describe your condition last month?

(1) Have felt quite calm and satisfied last month

(2) Has been somewhat plagued by agitation and nervousness last month

(3) Has been much bothered by agitation and nervousness last month

(4) Other/Wishes not stated

Have you experienced being depressed in the last month?

(1) Yes

(2) No

How have you experienced being depressed at the moment?

|  | Not experienced | To a small extent | To some extent | To a large extent | To a very large extent |
| --- | --- | --- | --- | --- | --- |
| Experience of sadness | (1) 🔾 | (2) 🔾 | (3) 🔾 | (4) 🔾 | (5) 🔾 |
| Tense and uneasiness | (1) 🔾 | (2) 🔾 | (3) 🔾 | (4) 🔾 | (5) 🔾 |
| Difficulty sleeping | (1) 🔾 | (2) 🔾 | (3) 🔾 | (4) 🔾 | (5) 🔾 |
| Difficulty concentrating | (1) 🔾 | (2) 🔾 | (3) 🔾 | (4) 🔾 | (5) 🔾 |
| Hard to get things done | (1) 🔾 | (2) 🔾 | (3) 🔾 | (4) 🔾 | (5) 🔾 |
| Negative thoughts/self-criticism | (1) 🔾 | (2) 🔾 | (3) 🔾 | (4) 🔾 | (5) 🔾 |
| Thoughts of ending life | (1) 🔾 | (2) 🔾 | (3) 🔾 | (4) 🔾 | (5) 🔾 |
| Have you experienced social anxiety in the past month? | (1) 🔾 | (2) 🔾 | (3) 🔾 | (4) 🔾 | (5) 🔾 |

Other, describe: _____

*Avoid sharing personally identifiable information to ensure your anonymity.*

Have you “killed” problems with drugs or other drugs in the last month?

(1) No

(2) To a small extent

(3) To some extent

(4) To a large extent

(5) To a very large extent

Thank you for your participation. Remember that your answers are anonymous, so we have no way of following you up based on this survey.

If you experience mental stress or strain, remember that you can get help:
*Kamerathjelpen Mannsforum, tel: 33 33 70 00
Norsk Folkehjelp, tel: 22 03 77 00
Kirkens SOS, tel: 23 08 13 80
Red Cross emergency, tel: 815 55 201
Mental Health, tel: 116 123*
